# Detection of Healthcare-Related Extended-Spectrum Beta-Lactamase-Producing *Escherichia coli* Transmission Events Using Combined Genetic and Phenotypic Epidemiology

**DOI:** 10.1371/journal.pone.0160156

**Published:** 2016-07-27

**Authors:** Anne F. Voor in ‘t holt, Agnes A. Wattel, Stefan A. Boers, Ruud Jansen, John P. Hays, Wil H. F. Goessens, Margreet C. Vos

**Affiliations:** 1 Department of Medical Microbiology and Infectious Diseases, Erasmus MC University Medical Center, Rotterdam, The Netherlands; 2 Department of Molecular Biology, Regional Laboratory of Public Health, Haarlem, The Netherlands; The University of Hong Kong, CHINA

## Abstract

**Background:**

Since the year 2000 there has been a sharp increase in the prevalence of healthcare-related infections caused by extended-spectrum beta-lactamase (ESBL)-producing *Escherichia coli*. However, the high community prevalence of ESBL-producing *E*. *coli* isolates means that many *E*. *coli* typing techniques may not be suitable for detecting *E*. *coli* transmission events. Therefore, we investigated if High-throughput MultiLocus Sequence Typing (HiMLST) and/or Raman spectroscopy were suitable techniques for detecting recent *E*. *coli* transmission events.

**Methods:**

This study was conducted from January until December 2010 at Erasmus University Medical Center, Rotterdam, the Netherlands. Isolates were typed using HiMLST and Raman spectroscopy. A genetic cluster was defined as two or more patients carrying identical isolates. We used predefined definitions for epidemiological relatedness to assess healthcare-related transmission.

**Results:**

We included 194 patients; strains of 112 patients were typed using HiMLST and strains of 194 patients were typed using Raman spectroscopy. Raman spectroscopy identified 16 clusters while HiMLST identified 10 clusters. However, no healthcare-related transmission events were detected. When combining data from both typing techniques, we identified eight clusters (*n* = 34 patients), as well as 78 patients with a non-cluster isolate. However, we could not detect any healthcare-related transmission in these 8 clusters.

**Conclusions:**

Although clusters were genetically detected using HiMLST and Raman spectroscopy, no definite epidemiological relationships could be demonstrated which makes the possibility of healthcare-related transmission events highly unlikely. Our results suggest that typing of ESBL-producing *E*. *coli* using HiMLST and/or Raman spectroscopy is not helpful in detecting *E*. *coli* healthcare-related transmission events.

## Introduction

For many years, the spread of microorganisms expressing extended-spectrum beta-lactamase (ESBL) genes was limited to those circulating in hospitals, with most isolates being *Klebsiella pneumoniae* [1]. Since the year 2000 however, there has been a sharp increase in the prevalence of ESBL-producing microorganisms worldwide, whereby *Escherichia coli* has replaced *K*. *pneumoniae* as the major carrier of ESBL encoding genes [2–6]. Worldwide, ESBL-producing Enterobacteriaceae carriage rates in the community range from <10% in Europe, to >50% in Southeast Asia, with *E*.*coli* as the predominant colonizing species [7, 8]. In addition, current research in the Netherlands shows that 5 to 7% of people in the Dutch community carry ESBL-producing *E*. *coli* isolates, with ST 131 being the most dominant sequence type [9–13]. *E*. *coli* is the most common agent associated with infections of the urinary tract and bloodstream infections arising from these urinary tract infections. Importantly, an increase in the number of antibiotic resistant bacteria in the community results in an increase in the number of antibiotic resistant bacteria in patients admitted to hospitals. The monitoring and prevention of healthcare-related infections requires the early detection and differentiation of antibiotic resistant bacteria in order to identify possible sources of transmission. This then allows suitable action to be taken in order to prevent future transmission events and infections [14].

Resistant isolates can be routinely compared to detect hospital transmission using molecular typing methods–with Pulsed-field gel electrophoresis (PFGE) as the gold standard technique. Although this technique has a good discriminatory power, it is too laborious to detect clones on a routine basis. Therefore, investigations need to be performed to determine whether high-throughput techniques such as High-Throughput Multilocus Sequence Typing (HiMLST) and Raman spectroscopy are suitable techniques to be implemented in a routine setting. HiMLST is a genotyping technique which classifies isolates based on the sequence variations of seven housekeeping genes. While conventional MLST uses classical Sanger sequencing, HiMLST employs next-generation sequencing (NGS) to generate MLST sequence data using a high–throughput protocol [15]. Raman spectroscopy is an easy to use and rapid technique which measures phenotypic expression profile differences between bacteria [16]. This publication investigated whether either of these techniques, or a combination of both, could be used to differentiate between recent ESBL-producing *E*. *coli* transmission events in hospitals by comparing the output of the techniques with patients’ hospital admission history.

## Methods

### Ethics statement

Written approval to conduct the study was received from the medical ethics research committee of the Erasmus MC University Medical Center (Erasmus MC), Rotterdam, the Netherlands (MEC-2011-085). As per conclusion of our medical ethics research committee, written informed consent from patients for acquiring data from clinical records was not needed. The existing data from the electronic medical records could not be recorded anonymously as the patient name is always visible when collecting data. Before data analyses however, the opt-out list available at our department was consulted and patients were excluded when applicable. Also before analyses, patient names were removed from the dataset by A.F. Voor in ‘t holt and A.A. Wattel. The authors had no direct interaction with the patients during the study period and no new patient data were collected for this study.

### Design and setting

For this retrospective study patients were included from January until December 2010 at Erasmus MC, Rotterdam, the Netherlands. In this university hospital all medical specialties are available. In 2010, 40,626 patients were admitted resulting in 292,209 admission days.

### Bacterial collection and patient data

ESBL-producing *E*. *coli* isolates (according to the Clinical and Laboratory Standards Institute (CLSI) criteria), were obtained from the bacterial biobanks of the Department of Medical Microbiology and Infectious Diseases (MMIZ) at Erasmus MC (frozen stocks stored at -80°C) [17]. Only the first *E*. *coli* isolate per patient was included in the study. All isolates were obtained from clinical samples sent to MMIZ for analysis due to: 1) an assumed infection, or 2) for surveillance purposes in the intensive care unit (ICU) and hematology departments of Erasmus MC. Patients admitted to ICU and hematology departments routinely receive selective digestive tract decontamination and are tested for the presence of (antibiotic resistant) Gram-negative bacteria twice weekly. If (antibiotic resistant) Gram-negative bacteria are detected, affected patients are immediately moved to ‘contact’ or ‘contact-droplet’ isolation in single-occupancy rooms. Healthcare workers wear gloves and gowns during patient care. No extra active surveillance and/or contact investigation was performed for this study.

Patient data (e.g. age, gender, and region of residence), hospital admission data (e.g. period, department, and patient room) and bacteriological data were obtained from electronic patient records. The age of the patient was defined as his/her age on the day of detection of the first ESBL-producing *E*. *coli* isolate. Mortality was defined as death from any cause within 28 days after the day of detection of the first ESBL-producing *E*. *coli* isolate. Twenty-eight-day mortality data was obtained from electronic patient records.

### High-throughput multilocus sequence typing

Due to availability and costs, a random selection of isolates were subjected to the standardized MultiLocus Sequence Typing (MLST) scheme for *E*. *coli* as reported by Wirth *et al*., 2006, using the High-Throughput MLST (HiMLST) strategy [15, 18]. PCR primers were modified to reduce amplicon sizes, conserving the intact cores, and extended with universal tails to make the isolates suitable for HiMLST use. The HiMLST primer sequences used in this publication are shown in Table 1. Advantages of using the HiMLST technique in comparison to conventional MLST is that it allows genotyping of large numbers of isolates, requires less labour per isolate, and lowers overall costs [15]. MLST types were generated using BioNumerics v6.6 software152 (Applied Math NV, Sint-Martens-Latem, Belgium), and by reference to the MLST database hosted at the University of Warwick (http://mlst.warwick.ac.uk/mlst/mlst/dbs/Ecoli).

**Table 1:** High-throughput MultiLocus Sequence Typing primer sequences used in this publication.

Gene	Forward primer	Reverse primer
adk	5'—gacactatagattctgcttggcgctccggg—3'	5'—cactatagggccgtcaactttcgcgtattt—3'
fumc	5'—gacactatagggtatttagtccagtac—3'	5'—cactatagggatttaggcttgttgtctg—3'
gyrb	5'—gacactatagataactcctataaagtgtc—3'	5'—cactatagggaatgttgttggtaaagcag—3'
icd	5'—gacactatagccagccatgctgaaagtg—3'	5'—cactatagggcaccagagtcacagagtc—3'
mdh	5'—gacactatagtgcacgaaccagagacag—3'	5'—cactatagggatgtcgttcttatctctgc—3'
pura	5'—gacactatagcatgtccgctgatccttg—3'	5'—cactatagggcggtcgggaacggacctgc—3'
reca	5'—gacactatagacctttgtagctgtaccacg—3'	5'—cactatagggagcgtgaaggtaaaacctgtg—3'

### Raman spectroscopy

Phenotypic relatedness was investigated via Raman spectroscopy using a SpectraCell*RA* (SC*RA*) apparatus (RiverD International B.V., Rotterdam, The Netherlands). Raman spectroscopy is a label-free, optical technology based on the inelastic scattering of light by molecules [19]. The Raman spectrum displays molecule-specific changes in wavelength—so-called spectroscopic fingerprints [19]. These fingerprints reflect the overall molecular composition of a sample [19]. The advantages of Raman spectroscopy are that this is an easy-to-use rapid technique. However, one disadvantage is that it is not currently a widely used technique. Cultures, sample preparation and SC*RA* measurements were performed according to the operator manual (version 1.7) [16]. Raman spectroscopy analyses and calculations were performed as described previously [19].

### Epidemiological relatedness

We defined a ‘cluster’ as two or more patients carrying identical isolates as indicated by HiMLST and/or Raman spectroscopic analysis. Within clusters, a ‘primary patient’ was defined as the first patient in time who was positive for an ESBL-producing *E*. *coli* isolate, while ‘secondary patients’ were all subsequent patients who were positive for an ESBL-producing *E*. *coli* isolate that was genetically or phenotypically related to the primary patient as identified by HiMLST and/or Raman spectroscopy. Non-cluster (unique) patients were defined as primary patients. The ‘transmission index’ was calculated for genotypically and phenotypically identical ESBL-producing *E*. *coli* isolates and was calculated as the number of secondary patients divided by the number of primary patients. To be able to distinguish community acquisition from healthcare-related transmission, we defined ‘healthcare-related transmission’ as ESBL-producing *E*. *coli* identified in a sample taken between 48 hours after admission and within 48 hours after discharge.

### Likelihood of healthcare-related transmission

To determine the likelihood of epidemiological relatedness of isolates within clusters we defined 4 groups based on the likelihood of healthcare-related transmission: 1) ‘definite’, 2) ‘probable’, 3) ‘possible’, and 4) ‘impossible’ (Table 2). Patients were ‘definitely related’ if patients shared the same patient room within the same admission period. If patients shared the same patient room but did not have the same admission period, and if the second patient was admitted within two months after the first patient was discharged, then patients were defined as ‘probably related’. ‘Possibly related’ patients only shared the same department during the same admission period. Alternatively, any second patient who was admitted within two months after their first patient was discharged was also defined as ‘possibly related’. Patients ‘impossibly related’ were patients related neither in place nor in time. When using these definitions we assumed 1) that patients were not mobile outside their original ward and 2) that people who were mobile between rooms and wards did not transmit the microorganism.

**Table 2:** Definitions of likelihood of epidemiological relatedness.

	Definition						
	Definite	Probable	Possible		Impossible		
**Same patient room**	1	1	0	0	0	0	0
**Same department**	1	1	1	1	0	1	0
**Same period**	1	0^a^	1	0^a^	1	0	0

Abbreviations: 0 = no; 1 = yes

^a^Not the same period but same patient room (probable) or department (possible) within 2 months after primary patient was discharged.

### Models of transmission

We compared different categories using 4 different models in order to determine the likelihood of healthcare-related transmission events (Table 2). In ‘model 1’, we combined hospital admission data of patients with isolates within the same cluster according to HiMLST. In ‘model 2’, we combined hospital admission data of patients with isolates within the same cluster according to Raman spectroscopy. In ‘model 3’ we combined 2 months of hospital admission data from patients with isolates within the same cluster and further sub-divided HiMLST clusters by adding data from Raman spectroscopy. ‘Model 4’ was identical to model 3, but the patient data collected was extended to 3 months.

### Statistical Analysis

A non-parametric test was performed using an independent samples median test in order to compare median days of stay in the hospital before the detection of an ESBL-producing *E*. *coli* between patients in clusters and non-cluster patients. A *P* value of <0.05 was considered statistically significant and the analysis was performed using IBM SPSS version 21 (SPSS Inc., Chicago, IL, USA).

## Results

### Selection of isolates and patients

**Fig 1:**
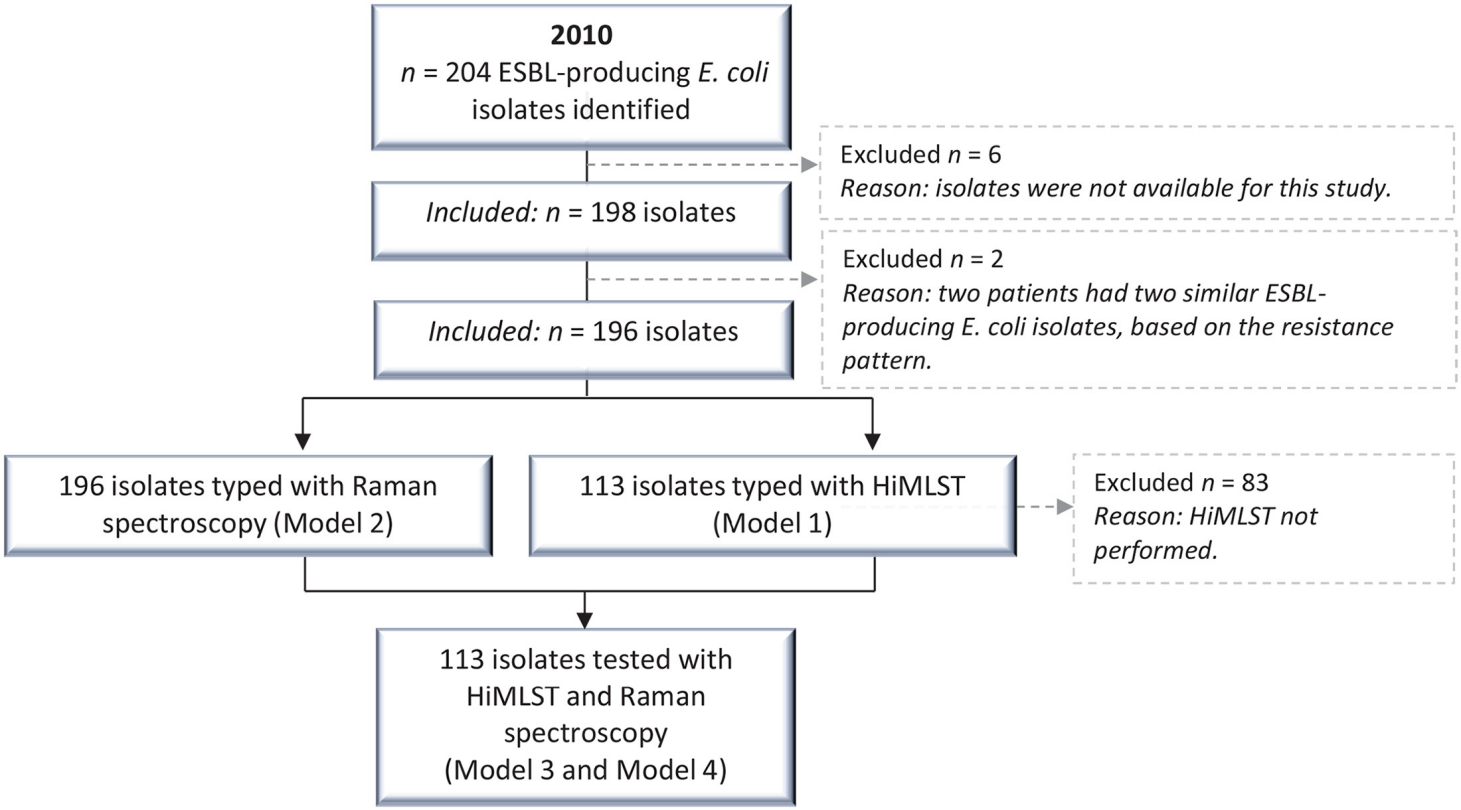
Flow diagram of the selection of isolates and patients identified with an ESBL-producing *E*. *coli* in 2010. The 113 isolates typed with HiMLST were a random selection out of the study total of 196 isolates. Abbreviations: HiMLST; High-throughput MultiLocus Sequence Typing, ESBL; extended-spectrum beta-lactamase.

In 2010, *E*. *coli* isolates were identified in 2,933 patients at Erasmus MC, including 204 patients (7.0%) with an ESBL-producing *E*. *coli*. The ESBL-rate per 1,000 hospital admissions was calculated as being 0.50. One hundred and ninety-eight ESBL-producing *E*. *coli* isolates from 194 patients were stored in MMIZ biobanks at Erasmus MC and were available for this study. We included 1 isolate per patient, except for 2 patients who were identified carrying 2 different ESBL-producing *E*. *coli* isolates. This difference was determined by differences in their antibiotic resistance profiles. In total, we included 196 isolates—of which 33 belonged to ST 131—representing 194 patients in the current study (Fig 1).

### Model 1

**Fig 2:**
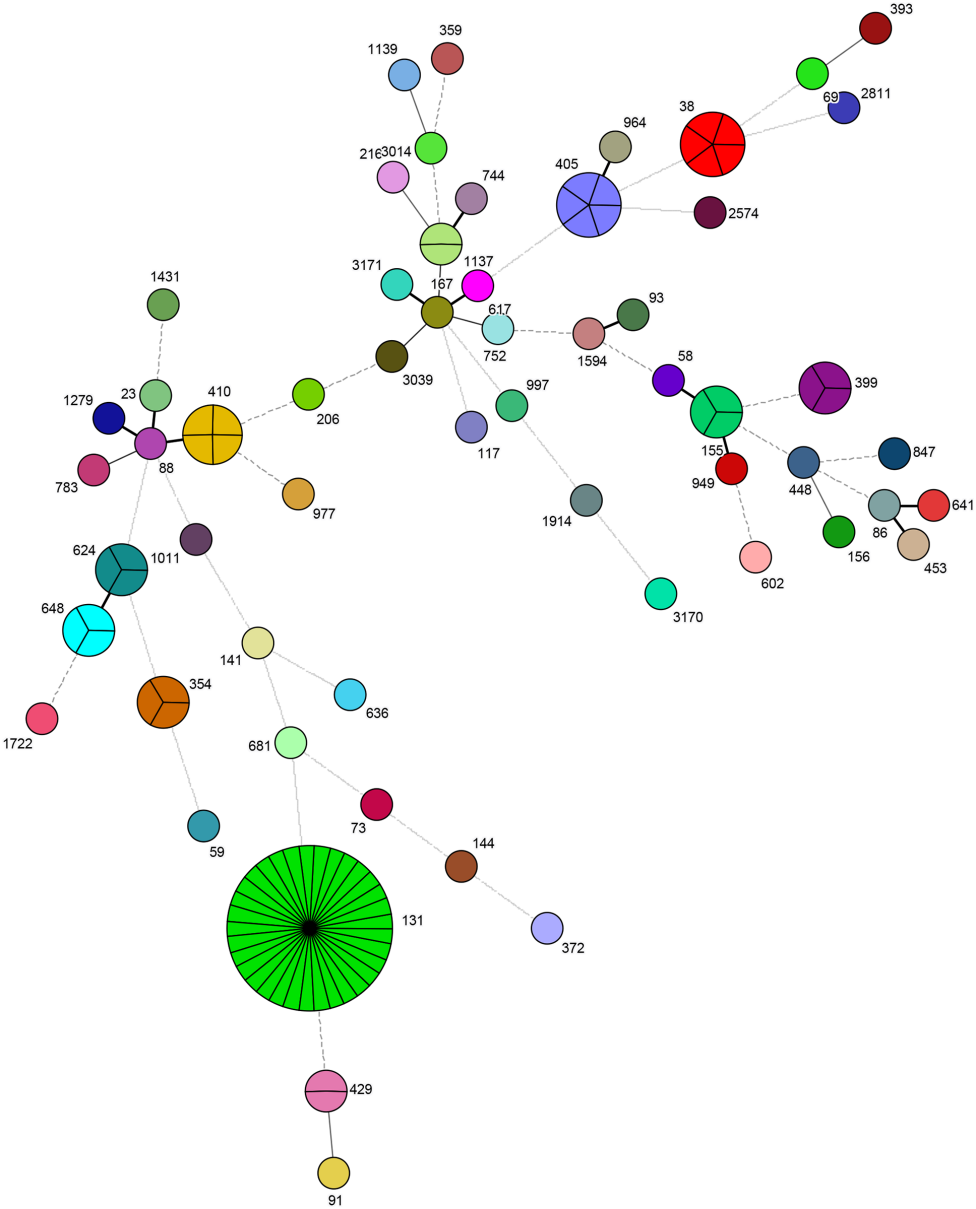
Representative genotypic ESBL-producing *E*. *coli* clusters observed using High-throughput multilocus sequence typing (HiMLST). The different colors represent different sequence types. 1 allele difference = thick solid line; 2 allele differences = medium solid line; 3 allele differences = thin solid line; 4 allele differences = dashed line; >4 allele differences = dotted line.

The first model included 113 randomly selected isolates (from 112 patients) typed using HiMLST (Figs 1 and 2). We identified 10 clusters (*n* = 65 isolates from 64 patients) with cluster size ranging from 2 to 33 patients—the largest cluster being ST 131, and 48 primary patients carrying a non-cluster, unique isolate ESBL (Table 3, Fig 2). After applying the definitions described in Table 2, we identified 3 possible healthcare-related transmission events within the cluster representing ST 131 (Table 3). All other patients were impossible to relate to each other with respect to time and place. For patients in the 10 clusters, 14 isolates (21.5%) were considered as healthcare-related transmission (Table 3) and the remaining isolates considered as community acquired. Of the 2 patients previously described carrying 2 different ESBL-producing *E*. *coli* isolates, the isolates of a single patient were typed using HiMLST. Results showed that these isolates had the same sequence type (ST 1137). This was the only patient present with this sequence type.

**Table 3:** Epidemiological relatedness of ESBL-producing *E*. *coli* isolates typed using HiMLST only.

Cluster no.	Sequence type	No. of patients	Healthcare-related^a^	Model 1			
				Definite	Probable	Possible	Impossible
1	38	5	3	0	0	0	5
2	131	33	3	0	0	3^b^	30
3	155	3	0	0	0	0	3
4	354	3	1	0	0	0	3
5	399	3	2	0	0	0	3
6	405	5	3	0	0	0	5
7	410	4	0	0	0	0	4
8	429	2	1	0	0	0	2
9	624	3	0	0	0	0	3
10	648	3	1	0	0	0	3
Total	n.a.	64	14	0	0	3	61

Abbreviations: HiMLST, High-throughput MultiLocus Sequence Typing; n.a., not applicable

^a^Positive isolate identified between 48 hours after admission and within 48 hours after discharge.

^b^Of which one isolate was considered as healthcare-related

### Model 2

The second model of this study included 196 isolates (from 194 patients) typed using Raman spectroscopy. We identified 16 clusters (*n* = 101 isolates*)*, with cluster sizes ranging from 2 to 26 patients, and 95 patients with a non-cluster isolate (Table 4). When applying the definitions as described in Table 2, only 1 patient was possibly related to another patient (Table 4). All other patients were impossible to relate with respect to time and place. From the patients in the 16 clusters, 33.7% (34 patients) were considered as originating from healthcare-related transmission events, and the remaining 66.3% considered as not originating from healthcare-related transmission events. For the 2 patients previously described with 2 different ESBL-producing *E*. *coli* isolates, both isolates from both patients showed different Raman spectroscopy cluster numbers.

**Table 4:** Epidemiological relatedness of ESBL-producing *E*. *coli* isolates typed using Raman spectroscopy only.

Cluster no.	No. of patients	Healthcare-related^a^	Model 2			
			Definite	Probable	Possible	Impossible
1	3	0	0	0	0	3
2	9	8	0	0	0	9
3	3	0	0	0	0	3
4	3	1	0	0	0	3
5	2	2	0	0	1	1
6	2	1	0	0	0	2
7	2	1	0	0	0	2
8	2	1	0	0	0	2
9	22	6	0	0	0	22
10	26	5	0	0	0	26
11	4	2	0	0	0	4
12	2	0	0	0	0	2
13	5	5	0	0	0	5
14	12	2	0	0	0	12
15	2	0	0	0	0	2
16	2	0	0	0	0	2
Total	101	34	0	0	1	100

^a^Positive sample identified between 48 hours after admission and within 48 hours after discharge.

### Model 3

The third model of this study included 113 isolates (from 112 patients) typed using both HiMLST and Raman spectroscopy. The median age of the 112 patients was 53.2 years (ranging from zero to 93) and 51 (45.5%) were male. The predominant sample site of the 113 isolates was urine (64.6%), followed by rectum and throat samples (15.0%; Table 5). Overall, 41.1% of patients lived in Rotterdam, the Netherlands. Data on city of residence was not available for 2 patients and only one patient lived abroad in Aruba (autonomy within the Kingdom of the Netherlands).

**Table 5:** Characteristics of patients (n = 112) and isolates (n = 113) typed using HiMLST in combination with Raman spectroscopy.

	No. of patients	Median age^a^ (range)	Male (%)	Crude Mortality (%)^b^	No. of isolates	Sample sites of isolates			
						Blood (%)	Urine (%)	Rectum/throat (%)	Other (%)^c^
Total	112	53.2 (0–93)	51 (45.5)	9 (8.0)	113	7 (6.2)	73 (64.6)	17 (15.0)	16 (14.2)
Cluster 1	4	51.8 (41–80)	1 (25)	1 (25)	4	0 (0)	4 (100)	0 (0)	0 (0)
Cluster 2	15	60.0 (2–86)	11 (73.3)	0 (0)	15	1 (6.7)	10 (66.7)	2 (13.3)	2 (13.3)
Cluster 3	3	45.7 (27–70)	1 (33.3)	0 (0)	3	0 (0)	2 (66.7)	0 (0)	1 (33.3)
Cluster 4	2	63.8 (62–66)	1 (50)	0 (0)	2	0 (0)	2 (100)	0 (0)	0 (0)
Cluster 5	2	68.3 (56–80)	1 (50)	0 (0)	2	0 (0)	1 (50)	0 (0)	1 (50)
Cluster 6	2	55.0 (39–71)	1 (50)	0 (0)	2	0 (0)	0 (0)	2 (100)	0 (0)
Cluster 7	3	86.4 (57–88)	3 (100)	1 (33.3)	3	0 (0)	1 (33.3)	2 (66.7)	0 (0)
Cluster 8	3	66.3 (47–69)	1 (33.3)	0 (0)	3	0 (0)	3 (100)	0 (0)	0 (0)
Non-cluster isolates	78	55.3 (0–93)	31 (39.7)	7 (9.0)	79	6 (7.6)	50 (63.3)	11 (13.9)	12 (15.2)

Abbreviations: HiMLST, High-throughput MultiLocus Sequence Typing.

^a^Age at day of detection of ESBL-producing *E*. *coli*

^b^Death from any cause within 28 days after first positive culture

^c^Including: pus, wound fluid, peritoneal cavity fluid

We identified 8 clusters when combining the results of Raman spectroscopy and HiMLST,—with cluster sizes ranging from 2 to 15 isolates (*n* = 34 patients, 30.4%), and 79 non-cluster isolates (78 patients, 69.6%; Table 5). This resulted in 86 primary patients (76.8%) and 26 secondary patients (23.2%), and a transmission index of 0.30. Of the 79 non-cluster isolates only 36 isolates (32.1%) were unique isolates according to both typing techniques, 11 isolates (9.8%) were part of a cluster according to Raman spectroscopy but not according to HiMLST, and 21 isolates (18.8%) were part of a cluster consistent with HiMLST but not consistent with Raman spectroscopy. In cluster 1 to 4, 100% of isolates belonged to ST 131 (Table 6), in cluster 5 to 8, no ST 131 isolates were identified.

**Table 6:** Epidemiological relatedness of ESBL-producing *E*. *coli* isolates typed using HiMLST in combination with Raman spectroscopy.

Cluster no.	No. of patients	Healthcare-related^a^	ST 131(%)	Model 3				Model 4			
				Definite	Probable	Possible	Impossible	Definite	Probable	Possible	Impossible
1	4	1	4 (100)	0	0	0	4	0	0	0	4
2	15	4	15 (100)	0	0	0	15	0	0	0	15
3	3	2	3 (100)	0	0	0	3	0	0	0	3
4	2	0	2 (100)	0	0	0	2	0	0	0	2
5	2	0	0 (0)	0	0	0	2	0	0	0	2
6	2	2	0 (0)	0	0	0	2	0	0	0	2
7	3	3	0 (0)	0	0	0	3	0	0	1	2
8	3	0	0 (0)	0	0	0	3	0	0	0	3
Total	34	12	24 (70.6)	0	0	0	34	0	0	1	33

Abbreviations: HiMLST, High-throughput MultiLocus Sequence Typing.

^a^Positive sample identified between 48 hours after admission and within 48 hours after discharge

Thirty-eight patients (33.9%) were detected with an ESBL-producing *E*. *coli* between 48 hours after admission and within 48 hours after discharge and were therefore considered as healthcare-related transmission events. The median length of stay in the hospital of these patients before detection was 11.5 days (ranging from three to 150 days). Of patients in clusters (*n* = 12), the median length of stay in the hospital before detection was 16.0 days (ranging from 3 to 49 days) and in patients with a non-cluster isolate (*n* = 26) the median length of stay in the hospital before detection was 11 days (ranging from 3 to 150 days) (*P* value 0.727). In total, 22 patients were identified with an ESBL-producing *E*. *coli* before admission, or within 48 hours after admission, and 52 patients had been discharged from hospital, or were outpatients, when the first positive culture was identified. These 74 patients were considered as having a community acquired ESBL-producing *E*. *coli*, though these 74 patients (66.1%) could still be a potential source of transmission events to other patients. After applying the definitions as described in Table 2, all patients were impossible to relate with respect to time and place (Table 6).

### Model 4

In model 4, we included 113 isolates (from 112 patients) that were typed using both HiMLST and Raman spectroscopy. This was identical to model 3, but patient data was collected over a 3 month period. Model 4 clusters and sizes were similar to those found in the previous word using model 3. After applying the definitions as described in Table 2, only 1 patient was possibly related to another patient. All other patients were impossible to relate in time and place (Table 6).

## Discussion

In this study, genetically and phenotypically defined clusters of ESBL-producing *E*. *coli* were identified using HiMLST and Raman spectroscopy, but no epidemiological relationships could be found between patients assigned to various epidemiological clusters of ESBL-producing *E*. *coli*. The most prevalent sequence type was ST 131 (33/113; 29.2%), which was expected since it is the most predominant sequence type circulating in the community in both The Netherlands and worldwide [20]. It was interesting that after sub-grouping the ST 131 isolates with Raman spectroscopy, 24/33 of these isolates could be subdivided into 4 different clusters, and 9 were considered as non-cluster (unique) isolates (model 3). However, despite this extra level of clustering, epidemiological relationships between these isolates and patients could still not be identified.

In outbreak settings, newer typing techniques such as whole genome sequencing (WGS) are proving to be helpful in healthcare- related transmission events settings, and are able to distinguish outbreak from non-outbreak bacterial strains [21, 22]. However, this technique still needs threshold analyses for defining recent transmissions. Also, currently, the WGS technique is not generally available for use in routine patient settings due to the fact that it is a complex, laborious, time-consuming and expensive technique.

In this publication, clinical and molecular epidemiology (both genetic and phenotypic) data have been combined in an attempt to detect healthcare-related transmission events in a non-outbreak setting. In the Netherlands, recently introduced guidelines for multidrug-resistant microorganisms stated that all ESBL-producing *E*. *coli* in Dutch hospitals should be typed in order to better detect and manage healthcare-related transmission events [23]. However, the exact definition of a ‘healthcare-related transmission event’ is not defined, and there are no defined typing techniques that are currently recommended for use. Additionally, it is not clear how any results obtained should actually be interpreted. Therefore, in this publication, the authors developed their own definitions for ‘healthcare-related transmission event’ and ‘likelihood of healthcare-related transmission’ (Table 2). In a systematic review, Kramer *et al*. determined how long *E*. *coli* can survive on inanimate surfaces, which differed from 1.5 hours up to 16 months [24]. As this range is not practical, we selected the reference that was most applicable to the hospital setting when considering environmental contamination in our definitions of epidemiological relatedness (Table 2). However, Neely at al. found that most *E*. *coli* isolates had died in the environment only after 36 days [25]. Therefore, we extended our time frame up to 2 months and incorporated this within the definitions ‘probable’ and ‘possible’ in Table 2. In case we still missed important links we extended the time frame used in model 3 from 2 to 3 months and used this time period in model 4 (Table 2). However, 33.9% of the 112 patients were considered as culture positive for ESBL-producing *E*. *coli* via a healthcare-related transmission event, but no healthcare-related transmission event was identified using our ‘likelihood’ definition. The question therefore arises if some of the 112 patients should actually be considered as ‘community acquired’, since there could still be some form of endogenous selection because of antibiotic use. This however is a subject for future research.

### Limitations

This study has some limitations. Firstly, the routine surveillance cultures in our dataset were obtained only from the adult ICU (3 different departments; 10 patients), children’s ICU (2 different departments; 2 patients) and hematology (1 department; 3 patients) while the remaining cultures were obtained from clinical samples. Therefore, the presence of unidentified ESBL-producing *E*. *coli* carrier patients cannot be ruled out. Also, the number of affected patients may have been underestimated, which would mean that the lack of epidemiological relatedness in this study could be a consequence of missing data. Secondly, we did not include the characterization of the specific ESBL genes (e.g. blaCTX-M, blaSHV, blaTEM) and plasmids in our analysis. It is known however that the IncFII plasmids harbor the CTX-M-15 enzyme, and that CTX-M-15 is mostly carried by the most prevalent ESBL-producing *E*. *coli* strain ST 131, which is also most prevalent in our study [26, 27]. Finally, the possibility of ESBL antibiotic resistance gene transmission between other members of the Enterobacteriaceae and our *E*. *coli* isolates was not investigated.

Though this publication suggests that determining ESBL-producing *E*. *coli* transmission events is difficult using currently available, and high throughput, typing technologies the spread of antibiotic resistant organisms within healthcare settings remains a serious problem. For example, the isolation of hospitalized patients with ESBL-producing *E*. *coli* is a nationwide policy in the Netherlands, and more studies are required in order to determine if, and when, contact isolation is required or no longer indicated. Interestingly, Tschudin-Sutter *et al*. showed that the rate of spread of ESBL-producing *E*. *coli* to roommates in hospitals was low and suggested discontinuing contact isolation of infected or colonized patients. However, these authors only included 93 patients in a study period of almost 12 years (June 1999 through April 2011) [8]. In any case, transmission prevention measures including antibiotic stewardship, cleaning and disinfection, barrier precautions and hand hygiene, should ideally be implemented in all healthcare settings [28, 29]. For example, Lautenbach *et al*. identified prior antibiotic usage as the only independent risk factor for acquiring an infection with ESBL-producing *E*. *coli* [28]. The fact that ESBL-producing *E*. *coli* transmission events are difficult to detect, means that the correct training of healthcare personnel in infection control procedures is extremely relevant. This in order to reduce the likelihood of transmission events occurring at all.

### Conclusion and clinical implication

ESBL-producing *E*. *coli* healthcare-related transmission events could not be successfully determined even when using predefined epidemiological definitions and both genotypic and phenotypic typing techniques (HiMLST and Raman spectroscopy). Even though the majority of isolates belonged to ST 131, no epidemiological relatedness was identified between patients carrying ST 131 *E*. *coli* strains. We therefore conclude that only the general use and development of more sensitive typing techniques (e.g. whole genome sequencing), coupled to increased throughput, will generate useful data for identifying ESBL-producing *E*. *coli* transmission events in healthcare environments. At the clinical level, the implementation of WGS should ideally be coupled to the screening of all patients at admission to hospitals as previously suggested [30].
